# A DFT Study of Band-Gap Tuning in 2D Black Phosphorus via Li^+^, Na^+^, Mg^2+^, and Ca^2+^ Ions

**DOI:** 10.3390/ijms252111841

**Published:** 2024-11-04

**Authors:** Liuhua Mu, Jie Jiang, Shiyu Gao, Xiao-Yan Li, Shiqi Sheng

**Affiliations:** 1 School of Physical Science and Technology, Ningbo University, Ningbo 315211, China; muliuhua@ucas.ac.cn (L.M.); jiangjie1@nbu.edu.cn (J.J.); 2 Wenzhou Institute, University of Chinese Academy of Sciences, Wenzhou 325001, China; 3 School of Physical Science, University of Chinese Academy of Sciences, Beijing 100049, China; 4 School of Physics, East China University of Science and Technology, Shanghai 200237, China; Y30221294@mail.ecust.edu.cn; 5 Department of Chemistry, Northwestern University, Evanston, IL 60208, USA; xiaoyan.li@northwestern.edu

**Keywords:** 2D black phosphorus, band gap, light metal ions, battery electrode, DFT calculations

## Abstract

Black phosphorus (BP) and its two-dimensional derivative (2D-BP) have garnered significant attention as promising anode materials for electrochemical energy storage devices, including next-generation fast-charging batteries. However, the interactions between BP and light metal ions, as well as how these interactions influence BP’s electronic properties, remain poorly understood. Here, we employed density functional theory (DFT) to investigate the effects of monovalent (Li^+^ and Na^+^) and divalent (Mg^2+^ and Ca^2+^) ions on the valence electronic structure of 2D-BP. Molecular orbital analysis revealed that the adsorption of divalent cations can significantly reduce the band gap, suggesting an enhancement in charge transfer rates. In contrast, the adsorption of monovalent cations had minimal impact on the band gap, suggesting the preservation of 2D-BP’s intrinsic electrical properties. Energetic and charge analyses indicated that the extent of charge transfer primarily governs the ability of ions to modulate 2D-BP’s electronic structure, especially under high-pressure conditions where ions are in close proximity to the 2D-BP surface. Moreover, charge polarization calculations revealed that, compared with monovalent cations, divalent cations induced greater polarization, disrupting the symmetry of the pristine 2D-BP and further influencing its electronic characteristics. These findings provide a molecular-level understanding of how ion interactions influence 2D-BP’s electronic properties during ion-intercalation processes, where ions are in close proximity to the 2D-BP surface. Moreover, the calculated diffusion barrier results revealed the potential of 2D-BP as an effective anode material for lithium-ion, sodium-ion, and magnesium-ion batteries, though its performance may be limited for calcium-ion batteries. By extending our understanding of interactions between ions and 2D-BP, this work contributes to the design of efficient and reliable energy storage technologies, particularly for the next-generation fast-charging batteries.

## 1. Introduction

Electrochemical energy storage devices, such as lithium-ion batteries (LIBs), sodium-ion batteries (SIBs), magnesium-ion batteries (MIBs), and calcium-ion batteries (CIBs), have seen rapid development [1,2,3,4,5]. However, at room temperature, the fast-charging capabilities of these devices are primarily limited by the reaction kinetics and structural stability of their active electrode materials [6,7]. Among the wide range of anode materials, graphite suffers from low capacity, insufficient safety, and poor performance under fast-charging conditions [8,9,10,11]. Similarly, silicon anodes are unsuitable for SIBs and potassium-ion batteries (PIBs) due to their significant volume changes during cycling [12,13]. Notably, phosphorus (P) anodes, particularly black phosphorus and its two-dimensional derivative exhibit remarkable potential due to their three-electron alloying reactions with metal ions M^+^ (P → MP → M_2_P → M_3_P), which yield a high theoretical specific capacity of 2590 mAh g⁻¹ and moderate redox potentials (~0.8 V vs. Li/Li^+^, and ~0.3 V vs. Na/Na^+^) [14,15,16,17]. Studies have demonstrated that M^+^ diffusion rates in BP anodes are superior to those in graphite, silicon, and other conventional anodes [1,18]. Moreover, BP exhibits anisotropic mechanical properties [19,20], a tunable direct band gap [21], and high carrier mobility [22], all of which could enhance its performance as an anode material in batteries. These intrinsic advantages have spurred great interest in utilizing P-based anodes for the next-generation fast-charging applications. Such applications necessitate direct interactions between lithium, sodium, magnesium, and calcium ions with the phosphorus anode, including the intercalation with ions during the charging process. From the perspective of chemistry, these interactions are often nonintuitive, as they strongly depend on external conditions and the surrounding chemical environment. Apart from a few experimental studies that have investigated the intercalation of lithium ions into black phosphorus [23,24], there remains a limited understanding of the interactions between a broader range of ions and black phosphorus, as well as their effects on the electronic properties of P-based anodes.

The interactions between nanoscale phosphorus structures and light metal ions cannot be adequately described by simple mathematical models derived from conventional chemical principles. In this context, numerical simulation techniques serve as a powerful and reliable tool to gain detailed physical insights into systems of interest. For instance, using DFT calculations, Colherinhas and co-workers investigated how monovalent and divalent ions affected the valence electronic structure of graphene via cation–π interactions [25]. Their results revealed that the lowest unoccupied molecular orbital (LUMO) of alkali ions (Li⁺, Na⁺) fits between the highest occupied molecular orbital (HOMO) and LUMO of graphene, thereby tuning the bottom of the conduction band and modulating the band gap. In contrast, Mg²⁺ ions share their orbitals with graphene, failing to modulate the band gap. To circumvent issues associated with infinite charged systems, they avoided periodic boundary conditions, opting instead for a graphene-like sheet model. Drawing inspiration from this work, we employed a 2D-BP sheet as a model to study the effects of monovalent and divalent ions (Li⁺, Na⁺, Mg²⁺, and Ca²⁺) on the valence electronic structure of 2D-BP via cation–π interactions. These interactions arise from the lone pair electrons on BP’s basal plane, which are evenly distributed to form a conjugated π-bond network [26] capable of interacting with metal ions.

In the present work, we found that the adsorption of divalent cations significantly reduced the band gap of 2D-BP, thereby suggesting its enhanced charge transfer rates. In contrast, monovalent cations exerted a minimal influence on the band gap, tending to preserve the electrical properties of 2D-BP. Further energetic and charge analyses revealed a partial charge transfer from 2D-BP to these divalent ions, indicating a robust cation–π interaction that significantly altered the electronic structure of 2D-BP, particularly under external pressure. Additionally, charge polarization calculations indicated that, when subjected to external pressure, the adsorption of divalent cations promoted greater polarizations in 2D-BP compared with monovalent cations, effectively breaking the symmetry of pristine 2D-BP. These findings highlight the critical role of ion adsorption in optimizing the electronic properties of BP, contributing to the development of more efficient and reliable energy storage technologies.

## 2. Results and Discussion

### 2.1. Molecular Orbital Analysis

**Figure 1:**
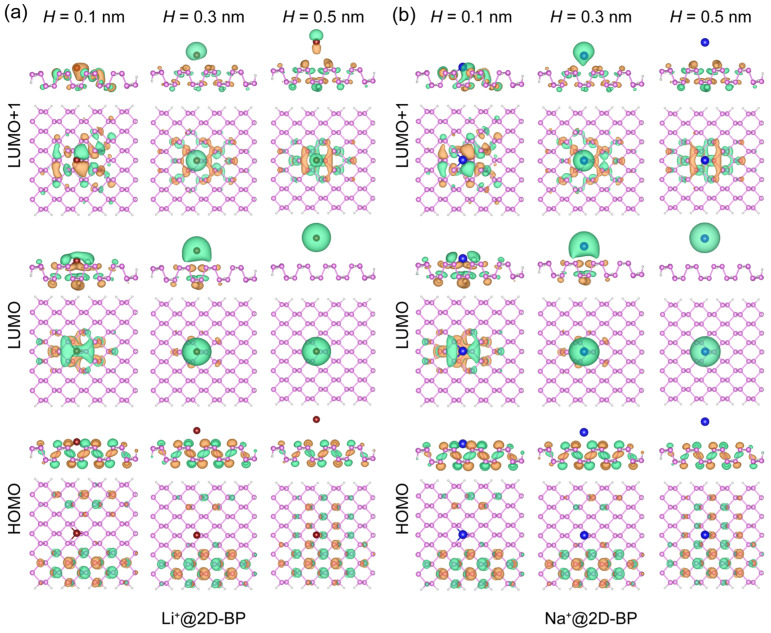
Spatial localization of frontier molecular orbitals in (**a**) Li^+^@2D-BP and (**b**) Na^+^@2D-BP complexes. The molecular orbital is plotted for iso-values of ±0.02 atomic units with orange and green denoting opposite signs. Spheres in red, blue, pink, and white represent Li^+^, Na^+^, phosphorus, and hydrogen, respectively.

Figure 1 illustrates the localization of the HOMO and LUMO of 2D-BP, as well as the LUMO of lithium and sodium ions. Following the approach used by Colherinhas and co-workers in their study on graphene systems [25], we assigned orbital attribution based on the spatial distribution of the molecular orbitals. For instance, in the frontier orbitals depicted in Figure 1a, the LUMO is primarily localized on the Li^+^, and we therefore refer to it as the LUMO of lithium. Conversely, the HOMO and LUMO+1 are predominantly localized on 2D-BP, leading us to designate them as the HOMO and LUMO of 2D-BP, respectively. Moreover, the small distances observed between 2D-BP and the ion (0.1–0.2 nm) can be interpreted as a result of high external pressure, a condition relevant to the ion-intercalation process during charging. These scenarios are of practical interest, as high pressure can significantly alter electronic energy levels and orbital localizations. For instance, decreasing the separation distance (denoted as *H*) between Li^+^ and 2D-BP by just 0.2 nm led to a notable change in orbital localization on the LUMO of the cation (Figure 1a). Specifically, in the Li^+^@2D-BP complex, at *H* = 0.1 nm, the LUMO of Li^+^ was significantly compressed by the 2D-BP surface and was partially shared with the 2D-BP sheet, compared with its distribution at *H* = 0.5 nm. For Na^+^, a similar monovalent ion, the LUMO in Figure 1b shows a more pronounced compression by the 2D-BP surface and greater sharing with the 2D-BP sheet at *H* = 0.1 nm compared with Li^+^. This observed difference between the Li^+^@2D-BP and Na^+^@2D-BP complexes (see more details in Figures S1 and S2) can be attributed to the variation in the van der Waals radius of these alkali cations, as the electron−electron repulsion in the system under pressure influences the valence bands [25]. Consequently, under pressure at *H* = 0.1 nm, Na^+^, with a larger van der Waals radius, experienced greater compression by the 2D-BP surface.

**Figure 2:**
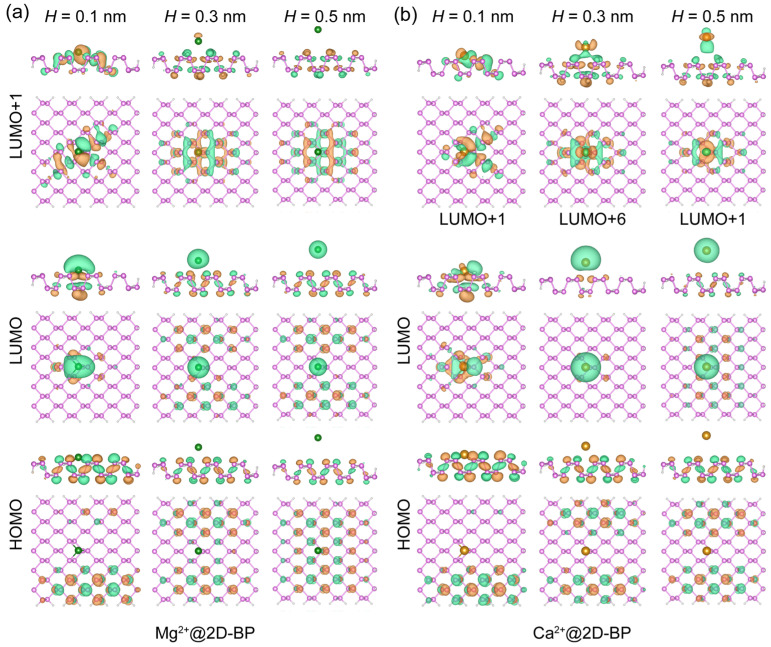
Spatial localization of frontier molecular orbitals in (**a**) Mg^2+^@2D-BP and (**b**) Ca^2+^@2D-BP complexes. The molecular orbital is plotted for iso-values of ±0.02 atomic units, with orange and green denoting opposite signs. Spheres in green, orange, pink, and white represent Mg^2+^, Ca^2+^, phosphorus, and hydrogen, respectively.

Next, we consider the behavior of the HOMO and LUMO of 2D-BP. For monovalent ions, under increased pressure (smaller *H* values), the spatial distribution of the HOMO on 2D-BP decreased, while in the case of the LUMO on 2D-BP (i.e., LUMO+1), greater pressure led to partial delocalization of the orbital onto the ion. This trend was observed for both Li^+^ and Na^+^, as shown in Figure 1. For divalent ions, as shown in Figure 2, the distribution of the LUMO for divalent ions was noticeably different from that of monovalent ions. At *H* = 0.5 nm, the LUMO of Mg^2+^ and Ca^2+^ partially delocalized onto the 2D-BP surface, a phenomenon not observed for Li^+^ and Na^+^. This indicates stronger binding in the complexes with divalent cations (see more details in Figures S3 and S4) and a greater degree of partial charge transfer from 2D-BP to the electron-deficient divalent cations. Furthermore, this distinction between divalent and monovalent ions likely arose from differences in their ability to modulate energy levels. Specifically, divalent cations significantly lowered the LUMO energy, bringing it closer to that of 2D-BP, and facilitating overlap or coupling between the orbitals. In contrast, monovalent ions had a less pronounced effect on the LUMO energy, which did not lead to noticeable orbital overlap or energy level alignment.

In addition to the distinct LUMO localization behavior of divalent ions with changing separation distance *H*, we observed unique behavior in the LUMO of 2D-BP for the Ca^2+^@2D-BP complex compared with the Li^+^@2D-BP, Na^+^@2D-BP, and Mg^2+^@2D-BP complexes. As shown in Figure 2b, increasing the distance between Ca^2+^ and 2D-BP by just 0.2 nm (from 0.1 nm to 0.3 nm) resulted in a sharp upward shift in the LUMO of 2D-BP. In the Ca^2+^@2D-BP complex, this orbital became the sixth molecular orbital above the LUMO. This was primarily due to the relatively low energies of the d-orbitals (Figure S5) in Ca^2+^, which filled the energy gap between the HOMO and LUMO of 2D-BP. However, at *H* = 0.5 nm, the LUMO of 2D-BP shifted back to the LUMO+1 of the entire complex, suggesting that even in the absence of external pressure (*H* = 0.3–0.5 nm), the hybridization between the d-orbitals of Ca^2+^ and the 2D-BP orbitals remained sensitive to the separation distance. Moreover, for the HOMO of 2D-BP, at *H* = 0.5 nm, no orbital occupation was observed beneath the Ca^2+^ ion in the Ca^2+^@2D-BP complex, while orbital occupation was present beneath the ions in the Li^+^@2D-BP, Na^+^@2D-BP, and Mg^2+^@2D-BP complexes. These differences highlight the distinct impacts that cations impose on the electronic properties of BP.

**Figure 3:**
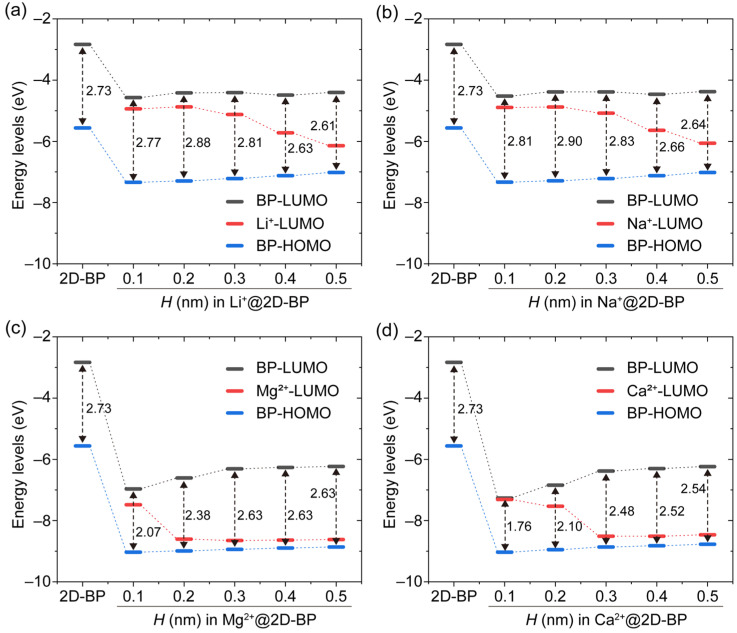
Evolution of frontier molecular orbital energy levels in (**a**) Li^+^@2D-BP, (**b**) Na^+^@2D-BP, (**c**) Mg^2+^@2D-BP, and (**d**) Ca^2+^@2D-BP complexes as a function of separation distance (*H*). Upon the introduction of a cation into the system, the energy levels of both the HOMO and LUMO of 2D-BP (denoted as BP-HOMO and BP-LUMO) underwent a significant decrease.

To evaluate the effect of metal ions on the electronic structure of 2D-BP, we plotted the evolution of frontier molecular orbital energy levels in the ion@2D-BP complexes (ion = Li^+^, Na^+^, Mg^2+^, and Ca^2+^) as a function of *H*, as shown in Figure 3. Overall, the adsorption of cations on 2D-BP effectively modulated the energy levels of both the LUMO and HOMO. The adsorption of divalent cations caused a significant reduction in the LUMO and HOMO energy levels of 2D-BP, by approximately 3 eV, which was notably larger than the reduction induced by monovalent cations (around 1.5 eV). Specifically, as the cations approached the 2D-BP, the LUMO energy level of 2D-BP in the Li^+^@2D-BP and Na^+^@2D-BP complexes fluctuated slightly around −4.4 eV, while the HOMO energy level gradually decreased. For the Mg^2+^@2D-BP and Ca^2+^@2D-BP complexes, the HOMO energy level of 2D-BP also decreased as the cations approached the 2D-BP. However, the LUMO energy level of 2D-BP behaved differently from that in the monovalent cation complexes. In the Mg^2+^@2D-BP complex, when *H* decreased from 0.5 nm to 0.3 nm, the LUMO energy level of 2D-BP slightly decreased from −6.23 eV to −6.31 eV, and as *H* further decreased to 0.1 nm, the energy level significantly dropped to −6.96 eV. For the Ca^2+^@2D-BP complex, the LUMO energy level of 2D-BP dramatically decreased from −5.88 eV to −7.27 eV as *H* decreased from 0.5 nm to 0.1 nm. These results indicate that while monovalent cations primarily influenced the HOMO energy level of 2D-BP, divalent cations had a more substantial effect on both the HOMO and LUMO energy levels. Notably, divalent cations exhibited strong control over the LUMO energy level of 2D-BP, consistent with the molecular orbital observations that the LUMO of divalent cations delocalized onto 2D-BP, while this behavior was absent with monovalent cations.

To further assess the effect of metal ions on the carrier dynamics of 2D-BP, we calculated the energy gap between the HOMO and LUMO orbitals, known as the HOMO–LUMO gap or band gap, which is an important factor in determining charge transfer rates. Generally, a smaller energy gap is associated with a higher charge transfer rate [27]. Here, the HOMO–LUMO gap was calculated at the B3LYP-D3/def2-TZVP level of theory. The HOMO–LUMO gap for the pristine 2D-BP sheet composed of 88 P atoms was 2.73 eV (Figure 3), which is slightly smaller than the previously reported value of 2.77 eV for a 2D-BP sheet with 84 P atoms at the B3LYP/DZP level of theory [28]. As the cations approached the 2D-BP, the HOMO–LUMO gap of 2D-BP in the Li^+^@2D-BP and Na^+^@2D-BP complexes increased from slightly below the value for the pristine 2D-BP sheet (about 2.6 eV) when *H* was greater than 0.3 nm, to slightly above the 2D-BP value (about 2.8 eV) when *H* was less than or equal to 0.3 nm (Figure 3a,b). These results suggest that the adsorption of monovalent cations has only a minor impact on the band gap of 2D-BP. However, this is beneficial for maintaining BP’s stability as an electrode material in LIBs and SIBs due to its unique electronic structure remaining largely preserved.

Figure 3c shows that the adsorption of Mg^2+^ at *H* = 0.1 nm significantly reduced the HOMO–LUMO gap to 2.07 eV compared with the pristine 2D-BP sheet (2.73 eV). As the separation between Mg^2+^ and 2D-BP increased, the HOMO–LUMO gap rose to 2.63 eV for the Mg^2+^@2D-BP complex at *H* = 0.5 nm. The Ca^2+^@2D-BP complex performed a similar trend, as shown in Figure 3d. Therefore, in addition to their strong binding to the π-electron system of 2D-BP, divalent cations effectively reduce the band gap of 2D-BP. Considering that a smaller band gap facilitates higher electron mobility at lower voltages, thereby reducing energy loss, divalent cations may enhance the performance of 2D-BP, and even bulk BP, as an electrode material in MIBs and CIBs under low-voltage conditions.

### 2.2. Mechanism Analysis

**Figure 4:**
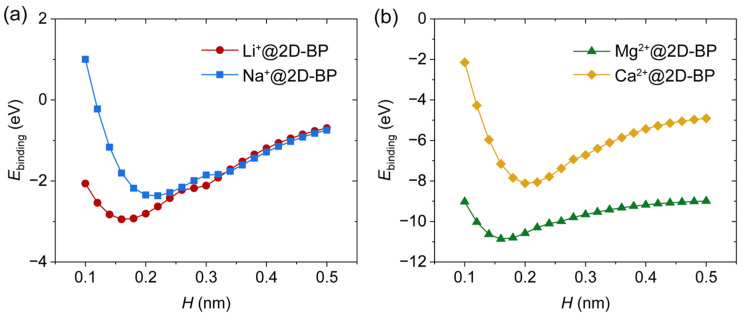
Binding energy of (**a**) Li^+^@2D-BP and Na^+^@2D-BP, as well as (**b**) Mg^2+^@2D-BP and Ca^2+^@2D-BP complexes as a function of separation distance (*H*).

**Figure 5:**
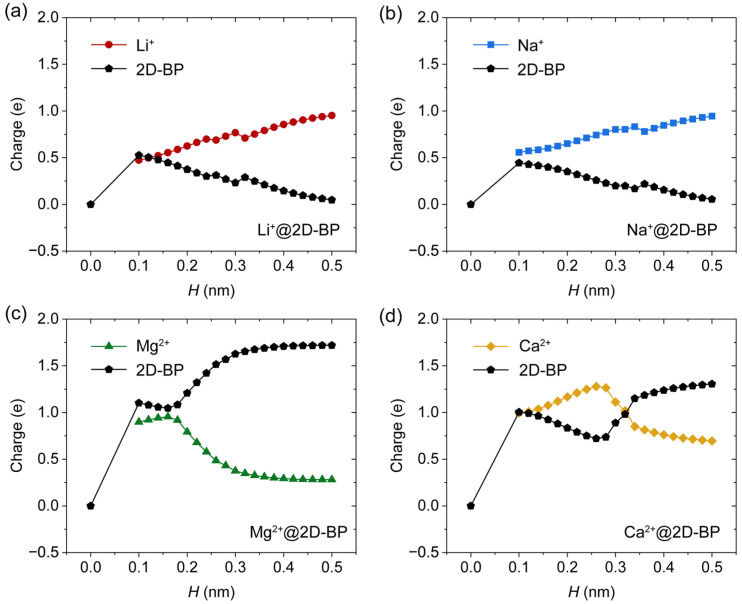
ADCH atomic charges on 2D-BP and the ion for (**a**) Li^+^@2D-BP, (**b**) Na^+^@2D-BP, (**c**) Mg^2+^@2D-BP, and (**d**) Ca^2+^@2D-BP complexes as a function of separation distance (*H*).

To investigate the physical nature of binding in the supramolecular complex and assess the relative binding strength of various ions, we calculated the binding energies between cations and 2D-BP (denoted as *E*_binding_), as well as the atomic dipole moment-corrected Hirshfeld (ADCH) charge distributions [29]. As shown in Figure 4a, the lowest binding energies for the Li^+^@2D-BP and Na^+^@2D-BP complexes were −2.95 and −2.37 kcal/mol, respectively. At the potential energy minimum, i.e., under zero external pressure, the linear distances between the cations and the nearest phosphorus atom in 2D-BP were 0.23 nm (Li^+^) and 0.27 nm (Na^+^). It is important to note that these distances are not the same as *H*. The covalent radius of phosphorus in 2D-BP was 0.11 nm, while the experimentally determined covalent radii of lithium and sodium are 0.13 and 0.15 nm, respectively [25]. Therefore, the ion–P distance of 0.23 versus 0.24 nm (Li^+^) and 0.27 versus 0.26 nm (Na^+^) correlate appropriately. If the interactions between alkali cations and the 2D-BP sheet were dominated by van der Waals forces, the calculated separation distances would be significantly larger. Furthermore, partial charge transfer (Figure 5a,b) was observed from 2D-BP to Li^+^ and Na^+^, especially when the ions were in close proximity to the 2D-BP surface. This indicates that the interaction is primarily governed by the well-known cation–π interaction, a relatively strong noncovalent interaction.

The binding of Mg^2+^ and Ca^2+^ to 2D-BP was approximately three to five times stronger than that of the alkali metal ions (Figure 4a,b). This level of binding energy is comparable to that of many single covalent bonds. A greater degree of partial charge transfer (Figure 5c,d) was observed from 2D-BP to Mg^2+^ and Ca^2+^, resulting in both species becoming more positively charged. This introduced a Coulomb repulsion component to the total complexation energy, which contributed to the unusual tail observed in the binding energy curves for Mg^2+^@2D-BP (0.2–0.5 nm) and Ca^2+^@2D-BP (0.3–0.5 nm). This tail reflects the fact that, even at large separation distances, the binding energy does not rapidly approach zero, and the atomic charges on 2D-BP do not return to neutral. A similar phenomenon has been observed in the adsorption of Mg^2+^ on graphene [25], which may be attributed to the limitations of DFT in accurately capturing long-range interactions, such as dispersion corrections. However, it is important to note that the failure of DFT to describe long-range interactions does not affect the conclusions of this study, as we focused on the potential energy minimum, where the trends in charge behavior for divalent ion complexes are consistent with those for monovalent ions.

The conclusions regarding energy levels were initially based on the hollow adsorption site (see Methods and Materials for more details). To further assess the effects of alternative sites (top and bridge), we analyzed ion adsorption at the potential energy minimum, examining the energy levels (Figure S6) and corresponding molecular orbitals (Figures S7 and S8). The results indicate that the overall trend in the HOMO-LUMO gap remained consistent across all adsorption sites. Notably, Na^+^ exhibited reduced sensitivity to site variations compared with the other cations (Figure S6), which may have contributed to more stable electronic properties during the diffusion of Na^+^. More importantly, the adsorption of divalent ions consistently caused a greater reduction in the band gap than monovalent ions, as seen in Figure 3, and this effect persisted across different adsorption sites.

Figure 5 illustrates that the charges on Li^+^, Na^+^, Mg^2+^, and Ca^2+^ deviated significantly from their nominal charges of +1e and +2e, respectively, with the degree of deviation depending on the proximity of the ion to the 2D-BP surface. The smaller the ion, the larger the amount of electronic charge it acquired from 2D-BP at covalent-distance separations. These significant alterations in the electronic population upon complexation suggest a strong polarizing effect exerted by each cation. Due to the delocalization (high potential energy) of phosphorus electrons, 2D-BP was highly responsive to such polarization effects.

**Figure 6:**
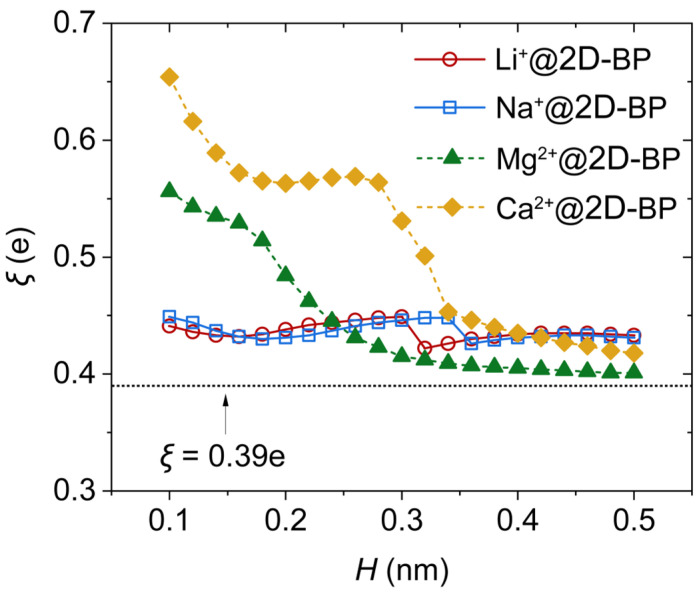
Polarizing action of an ion adsorbed on the 2D-BP sheet, expressed as $\xi ={\left(\sum_{i=1}^{N}q_{i}^{2}\right)}^{1/2}$, where $q_{i}$ represents the partial charges on each atom in the 2D-BP sheet. The dotted horizontal line corresponds to *ξ* of the isolated 2D-BP sheet.

The polarization of 2D-BP by an ion can be effectively quantified by examining the alteration of partial atomic charges in pristine 2D-BP. Following the approach used by Colherinhas and co-workers in their study on graphene systems [25], we calculated an integral measure of polarization, ξ, using the sum of squared ADCH atomic charges localized on each atom of the 2D-BP sheet (Figure 6). The non-zero value (0.39e) of *ξ* for the isolated BP sheet indicates the influence of edge hydrogen atoms. Specifically, these terminating hydrogen atoms provided a significant contribution to *ξ*, leading to the polarization of the isolated 2D-BP sheet, although they did not directly affect band-gap tuning. Consequently, *ξ* was systematically greater than zero, consistent with results observed for the graphene sheet, where hydrogen also contributed to the polarization of the graphene sheet [25]. Moreover, a remarkable observation was that ξ was highly sensitive to the presence and nature of the ion. Specifically, ξ remained relatively insensitive to the ion’s distance from the 2D-BP surface in the case of monovalent ions but was highly responsive to the distance for divalent ions. This is consistent with the molecular orbital analysis, which showed that divalent ions had a greater effect on the band gap of 2D-BP compared with monovalent ions.

It is worth noting that in most ion-containing systems, the value of ξ is larger than that of pure 2D-BP, indicating a strong polarizing effect exerted by each cation. In particular, when divalent ions are in close proximity to the 2D-BP surface, ξ values are significantly higher compared with monovalent ions, suggesting that divalent ions promote greater polarization in 2D-BP. This enhanced polarization contributes to breaking the symmetry of pristine 2D-BP, further reinforcing the stronger influence of divalent ions on 2D-BP’s electronic structure compared with their monovalent counterparts.

### 2.3. Diffusion Barrier

**Figure 7:**
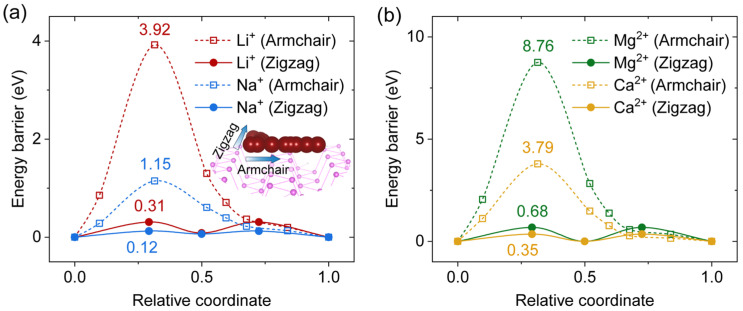
Diffusion energy barriers for (**a**) Li^+^/Na^+^ and (**b**) Mg^2+^/Ca^2+^ ions on a 2D-BP sheet along two diffusion paths, armchair and zigzag, as illustrated in the inset of panel (**a**). The diffusion calculations were based on the respective separation distances at the potential energy minimum.

Understanding the diffusion energy barriers is essential for evaluating battery performance. While previous theoretical studies have computed diffusion barriers for neutral metal atoms on 2D-BP surfaces [30,31], theoretical calculations for ion diffusion barriers remain scarce. This gap is critical, especially considering that ion intercalation on phosphorus anodes occurs during the charging process. To address this, we calculated the diffusion energy barriers for Li⁺, Na⁺, Mg²⁺, and Ca²⁺ ions on a 2D-BP sheet based on their separation distances at the potential energy minimum. Notably, all ions exhibited strong anisotropic diffusion behavior, with barriers along the zigzag direction being significantly lower than those along the armchair direction (Figure 7). This trend aligns with literature findings for neutral metal atoms on BP surfaces [30,31].

As expected, the diffusion barriers for monovalent ions (Li^+^ and Na^+^) along the zigzag direction (Figure 7a) were lower than those for divalent ions (Mg^2+^ and Ca^2+^, Figure 7b). Specifically, Na^+^ displayed the lowest diffusion barrier of 0.12 eV, even lower than that of Li^+^ (0.31 eV), which is consistent with results for neutral atoms [32]. This finding suggests that sodium-ion batteries based on 2D-BP could offer a higher rate capability compared with lithium-ion batteries. Interestingly, Mg^2+^ ions exhibited a diffusion barrier of 0.35 eV, comparable to that of Li^+^ (0.31 eV), indicating that Mg^2+^ ions could achieve favorable diffusion rates in 2D-BP anodes. In contrast, the diffusion barrier for Ca^2+^ ions was significantly higher at 0.68 eV, suggesting limited diffusion performance. Overall, these findings support the potential of 2D-BP as an effective anode material for lithium-ion, sodium-ion, and magnesium-ion batteries, though its performance may be limited for calcium-ion batteries.

## 3. Methods and Materials

All calculations were carried out using the ORCA quantum chemistry package (version 4.2.1) [33,34], employing the B3LYP functional [35] with the def2-TZVP basis set. Grimme’s DFT-D3 correction method [36] was applied to account for dispersion corrections, and thus this approach is referred to as B3LYP-D3. Basis set superposition errors (BSSEs) were corrected by using the geometric counterpoise (gCP) correction method [37]. The 2D-BP sheet model consisted of 88 phosphorus atoms and 26 hydrogen atoms. Here, the hydrogen atoms were used to passivate the dangling bonds of the 2D black phosphorus sheets, resulting in a closed-shell and neutral system. The positions of the hydrogen atoms were established by constructing multiple initial models with varying orientations, followed by structural optimization to identify the most stable configuration, which was then used for ion-adsorption calculations. The total charge of each system containing monovalent and divalent cations was +1e and +2e, respectively. For binding energy calculations, the x and y coordinates of the ions for this adsorption position were obtained from the optimized structure of the ion@2D-BP system at the hollow site, while the z coordinates were systematically scanned from 0.1 to 0.5 nm in increments of 0.02 nm to evaluate the distance from the 2D-BP substrate. Subsequently, single-point energy calculations were performed to obtain the total energies of the ion@2D-BP complexes at the various z coordinates. The binding energy (*E*_binding_) was defined as *E*_binding_ = *E*(ion@2D-BP) − *E*(2D-BP) − *E*(ion), where *E*(ion@2D-BP), *E*(2D-BP), and *E*(ion) are the total energies of ion@2D-BP complexes, the 2D-BP sheet, and a single ion, respectively. All single-point energies and optimizations were carried out using tight SCF (10^−7^ *E*_h_) convergence criteria. The polarizing effect of the ions is described by the function, $\xi ={\left(\sum_{i=1}^{N}q_{i}^{2}\right)}^{1/2}$, where *q*_i_ represents the partial charges on each atom in the 2D-BP sheet, and *N* = 114. Atomic dipole moment corrected Hirshfeld population (ADCH) charge analyses [38] were performed using the Multiwfn software (Version 3.8) [39]. ADCH analysis is widely used for systems dominated by noncovalent interactions [40,41,42].

## 4. Conclusions

In summary, this DFT study systematically explored how the interactions between metal ions (Li⁺, Na⁺, Mg²⁺, and Ca²⁺) and 2D-BP can influence the electronic properties of 2D-BP, particularly the band gap. Our findings, based on molecular orbital analysis and energetic and charge analyses, as well as charge polarization calculations reveal that divalent ions, such as Mg²⁺ and Ca²⁺, exhibit stronger binding interactions with 2D-BP compared with monovalent cations (Li⁺, and Na⁺), resulting in significant reduction in the band gap. The partial charge transfer from 2D-BP to these divalent ions, coupled with their pronounced orbital overlap, reveals a robust cation–π interaction [43,44,45] that significantly alters the electronic structure of 2D-BP, especially under external pressure. Given that a smaller band gap can enhance electron mobility at lower voltages and reduce energy losses, divalent ions may improve the performance of 2D-BP, and even bulk BP, as an electrode material in MIBs and CIBs at low operating voltages. Although the adsorption of monovalent ions on the 2D-BP surface does not significantly modulate its band gap, the preserved unique electronic structure of 2D-BP is still advantageous for maintaining stable performance when used as electrode material in LIBs and SIBs. The calculated diffusion barrier results support the potential of 2D-BP as an effective anode material for LIBs, SIBs, and MIBs, though its performance may be limited for CIBs.

The importance of phosphorus anodes in energy storage systems, especially for next-generation fast-charging applications, has been increasingly recognized due to their high theoretical capacity and favorable electrochemical properties [14,15,16,17]. The unique three-electron alloying reactions between phosphorus and metal ions provide a distinct advantage over conventional anode materials such as graphite and silicon [1,18]. Our study contributes a molecular-level understanding of the interactions between lithium, sodium, magnesium, and calcium cations with phosphorus anodes, which is critical for optimizing the performance of metal-ion batteries. The ability to fine-tune the electronic properties of BP through ion adsorption presents a promising approach for optimizing battery design. In particular, our findings may pave the way for the design of anodes with higher charge transfer rates, and enhanced capacity retention under fast-charging conditions. Moreover, by demonstrating how metal ions affect the valence electronic structure of BP, this study opens new possibilities for tailoring other anode materials to meet the growing demand for high-performance energy storage solutions in future batteries.

## Data Availability

The original contributions presented in the study are included in the article/Supplementary Materials. Further inquiries can be directed to the corresponding author.

## Supplementary Materials

The following supporting information can be downloaded at: https://www.mdpi.com/article/10.3390/ijms252111841/s1.
