# Supplementary material for: A DFT Study of Band-Gap Tuning in 2D Black Phosphorus via Li+, Na+, Mg2+, and Ca2+ Ions
Source: Int J Mol Sci. 2024 Nov 4;25(21):11841. doi: 10.3390/ijms252111841 (PMC11545926; doi:10.3390/ijms252111841)
Supplement: Supplementary file 1 [file ijms-25-11841-s001.zip › ijms-3295616-supplementary.pdf]

Supplementary material for

A DFT Study of Band Gap Tuning in 2D Black Phosphorus via Li^+, Na^+, Mg^{2+}, Ca^{2+} Ions

Liuhua Mu[1,2,3,†], Jie Jiang[1,†], Shiyu Gao[4], Xiao-Yan Li[5], and Shiqi Sheng[4,*]

[1] *School of Physical Science and Technology, Ningbo University, Ningbo 315211, China*; jiangjie1@nbu.edu.cn

[2] *Wenzhou Institute, University of Chinese Academy of Sciences, Wenzhou, Zhejiang 325001, China*; muliuhua@ucas.ac.cn

[3] *School of Physical Science, University of Chinese Academy of Sciences, Beijing 100049, China*

[4] *School of Physics, East China University of Science and Technology, Shanghai 200237, China*;
Y30221294@mail.ecust.edu.cn; shengshiqi@ecust.edu.cn

[5] *Department of Chemistry, Northwestern University, Evanston, IL 60208, United States*; xiaoyan.li@northwestern.edu

† These authors contributed equally to this work.

* Correspondence: shengshiqi@ecust.edu.cn

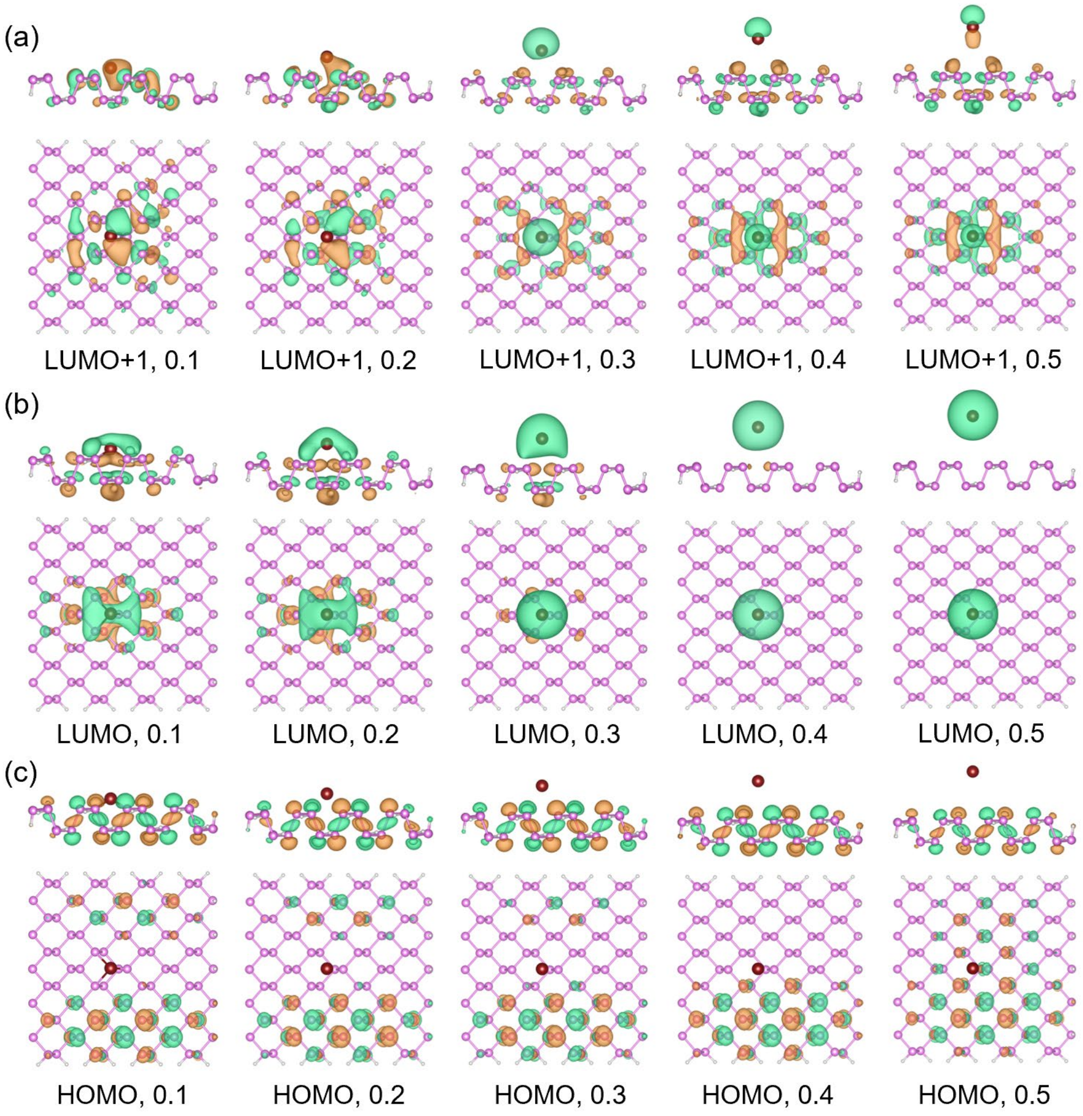

Figure S1. Spatial localization of frontier molecular orbitals for (a) LUMO+1 (b) LUMO, and (c) HOMO in Li^{+}@2D-BP complex at varying separation distances (*H*, in nm). The molecular orbital is plotted for iso-values of ±0.02 atomic units with orange and green denoting opposite signs. Spheres in red, pink, and white represent Li^{+}, phosphorus, and hydrogen respectively.

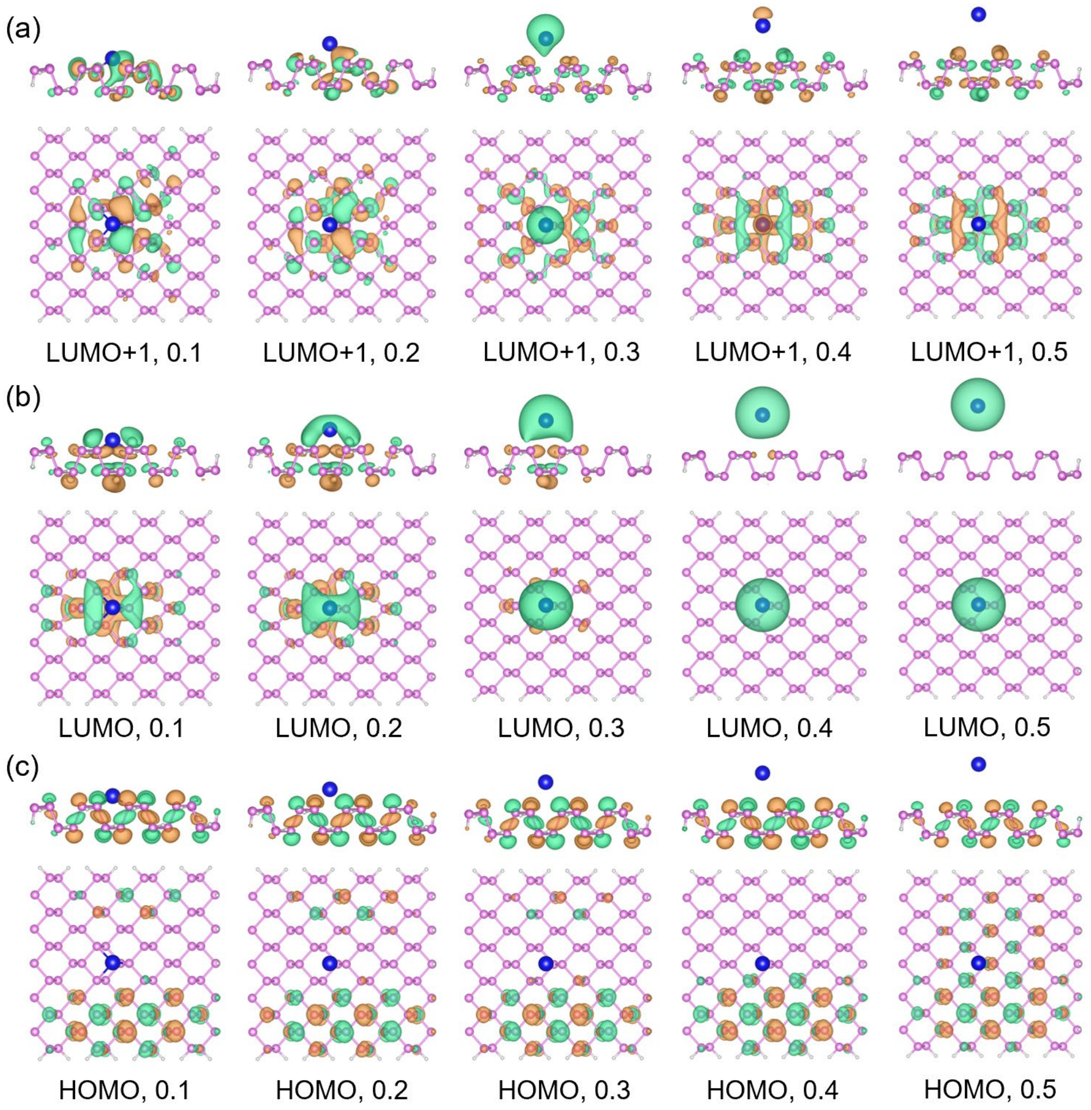

Figure S2. Spatial localization of frontier molecular orbitals for (a) LUMO+1 (b) LUMO, and (c) HOMO in Na^+@2D-BP complex at varying separation distances (*H*, in nm). The molecular orbital is plotted for iso-values of ±0.02 atomic units with orange and green denoting opposite signs. Spheres in blue, pink, and white represent Na^+, phosphorus, and hydrogen respectively.

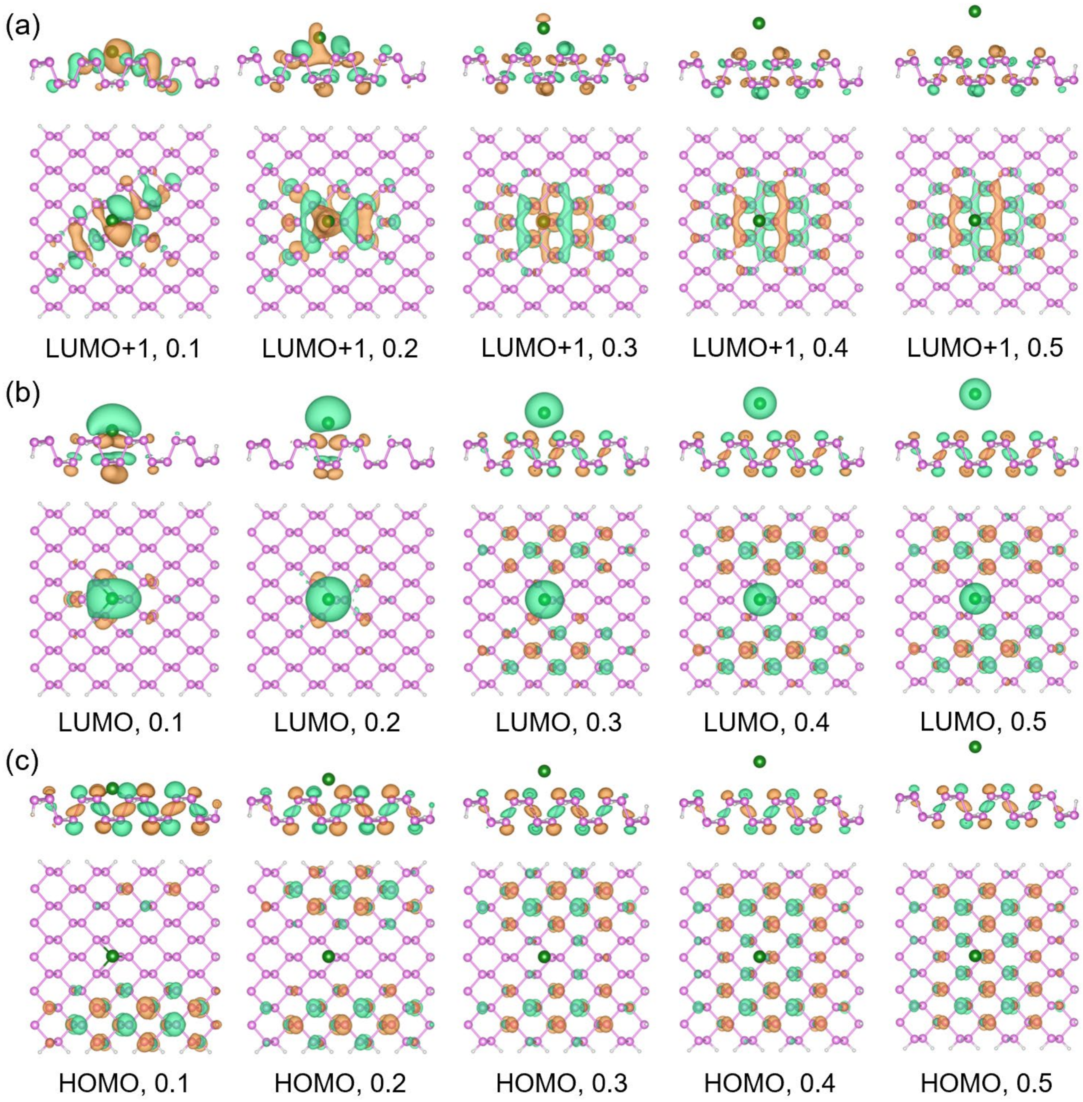

Figure S3. Spatial localization of frontier molecular orbitals for (a) LUMO+1 (b) LUMO, and (c) HOMO in Mg^{2+}@2D-BP complex at varying separation distances (*H*, in nm). The molecular orbital is plotted for iso-values of ±0.02 atomic units with orange and green denoting opposite signs. Spheres in green, pink, and white represent Mg^{2+}, phosphorus, and hydrogen respectively.

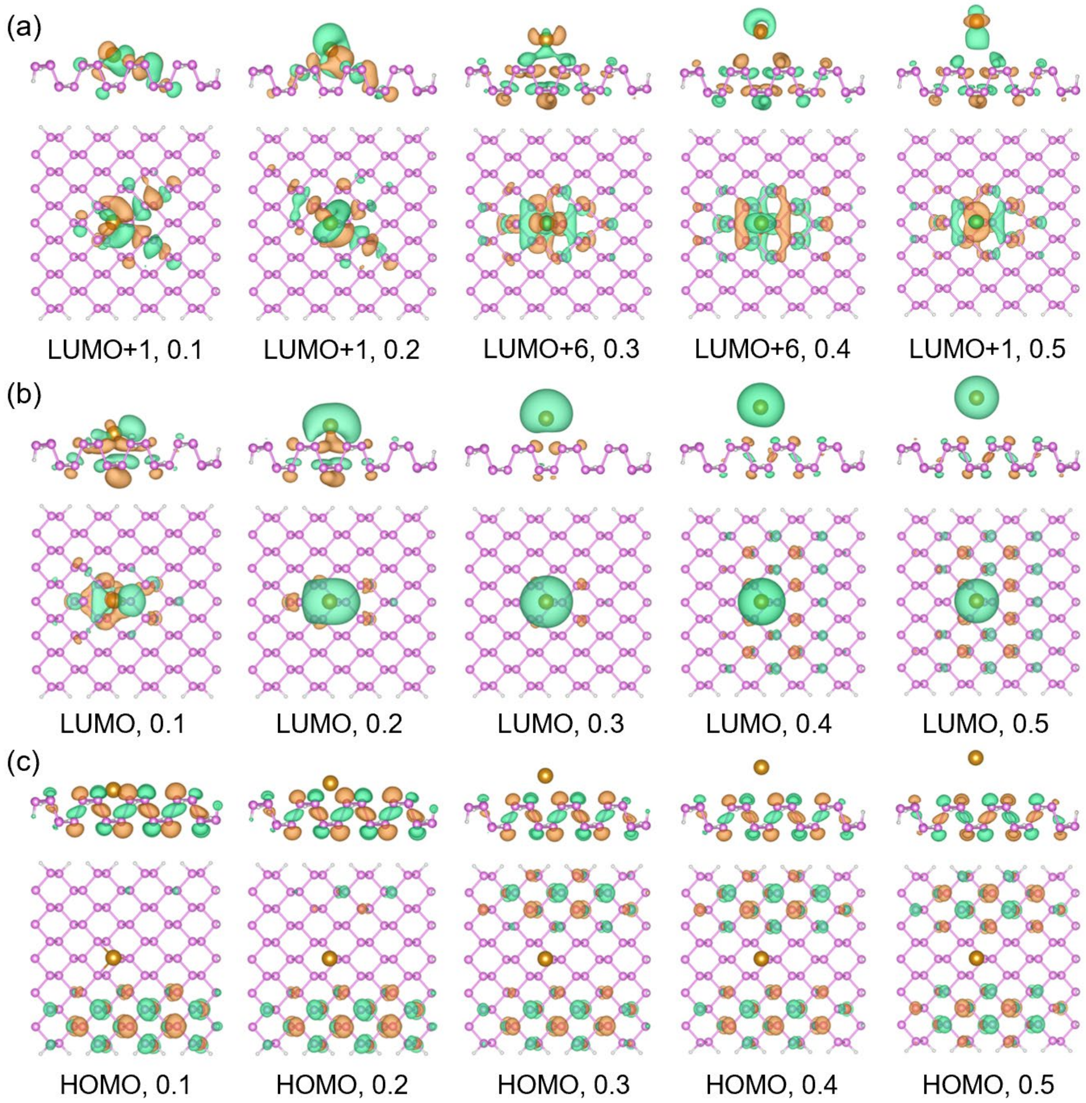

Figure S4. Spatial localization of frontier molecular orbitals for (a) LUMO+1/LUMO+6 (b) LUMO, and (c) HOMO in Ca^{2+}@2D-BP complex at varying separation distances (*H*, in nm). The molecular orbital is plotted for iso-values of ±0.02 atomic units with orange and green denoting opposite signs. Spheres in orange, pink, and white represent Ca^{2+}, phosphorus, and hydrogen respectively.

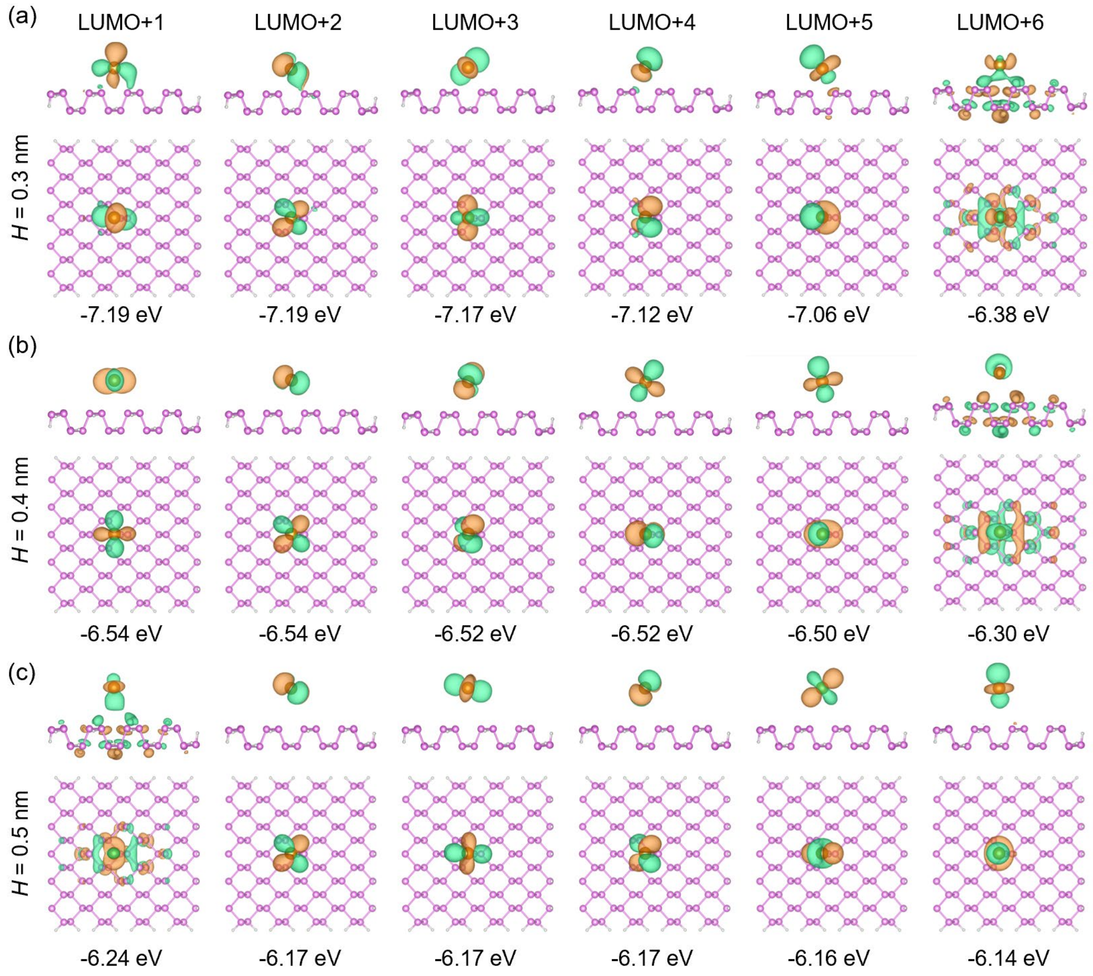

Figure S5. Spatial localization of frontier molecular orbitals in Ca^{2+}@2D-BP complex at larger separation distances: (a) H = 0.3 nm, (b) H = 0.4 nm and (c) H = 0.5 nm. Corresponding energy levels are provided below each image. All orbitals predominantly localized on the ion correspond to the d-orbitals of Ca^{2+}. The molecular orbital is plotted for iso-values of ±0.02 atomic units with orange and green denoting opposite signs. Spheres in orange, pink, and white represent Ca^{2+}, phosphorus, and hydrogen respectively.

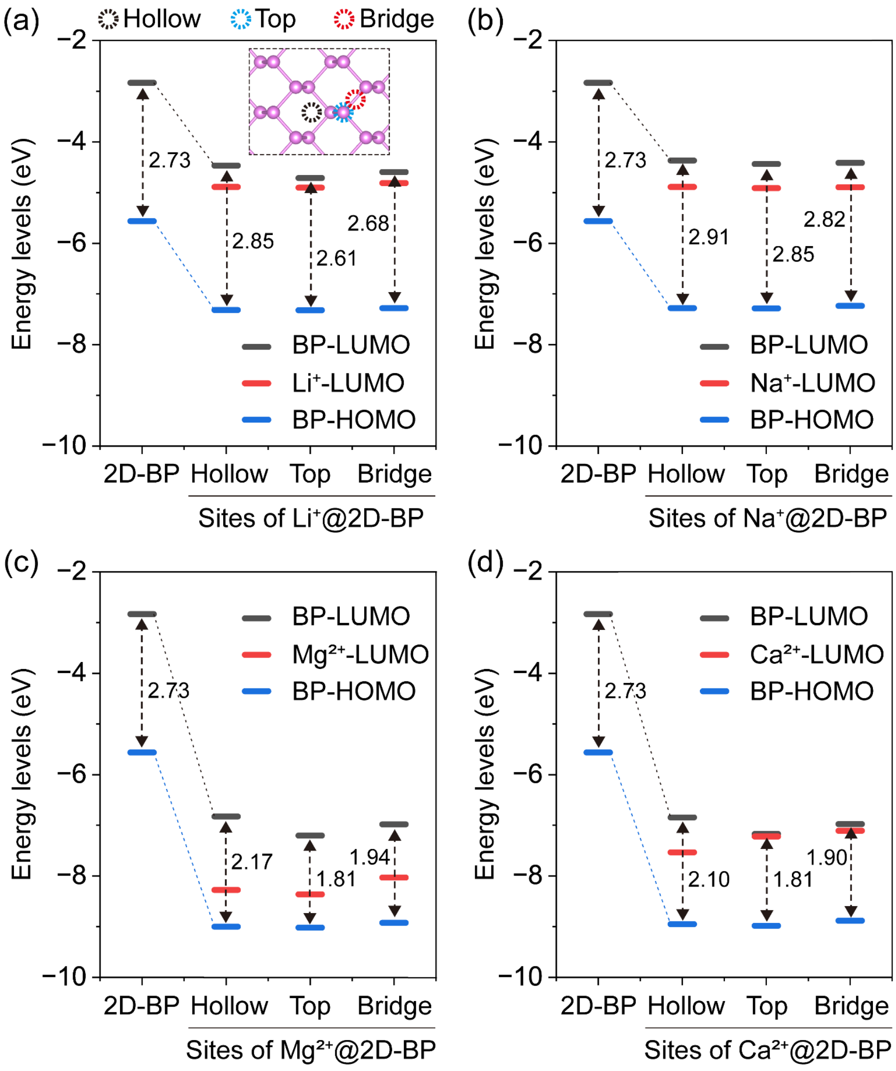

Figure S6. Evolution of frontier molecular orbital energy levels in (a) Li^+@2D-BP, (b) Na^+@2D-BP, (c) Mg^{2+}@2D-BP, and (d) Ca^{2+}@2D-BP complexes at the potential energy minimum across different adsorption sites (hollow, top, and bridge, as illustrated in (a)). Upon the adsorption of a cation into the system, the energy levels of both the HOMO and LUMO of 2D-BP (denoted as BP-HOMO and BP-LUMO) undergo a significant decrease.

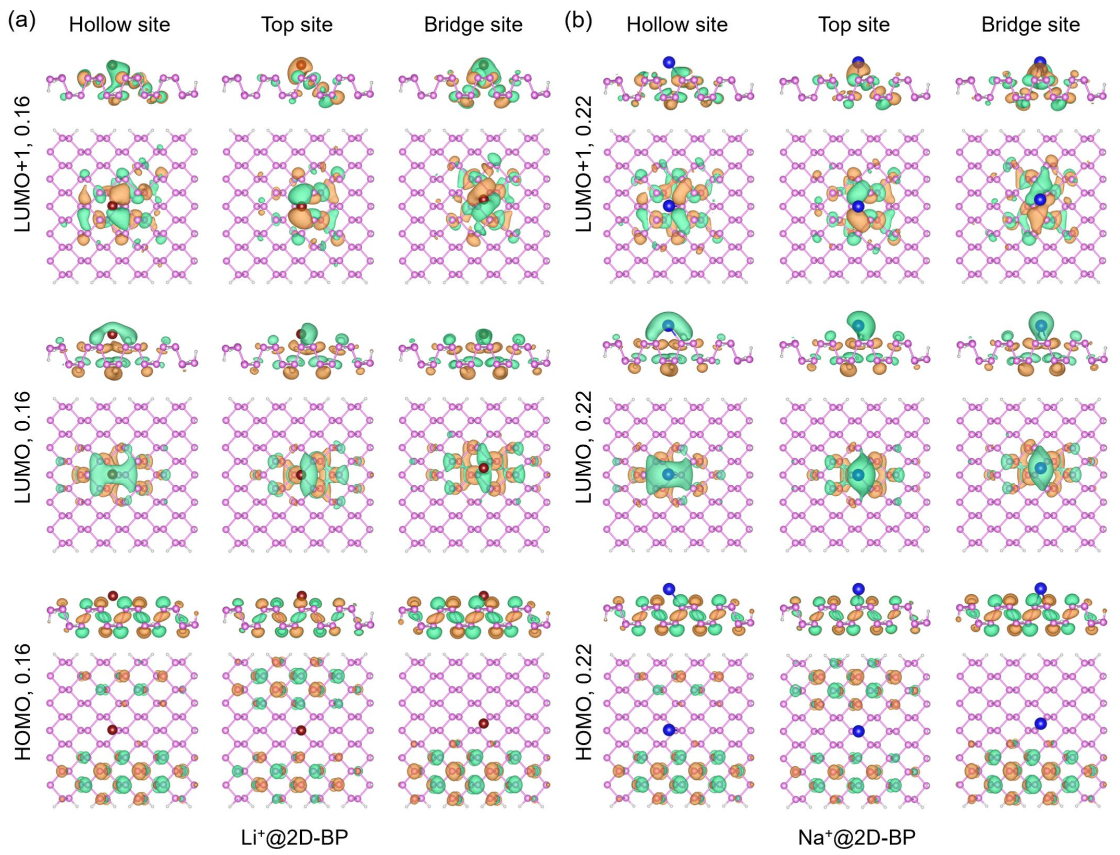

Figure S7. Spatial localization of frontier molecular orbitals in (a) Li^+@2D-BP and (b) Na^+@2D-BP complexes at the potential energy minimum across different adsorption sites (hollow, top, and bridge). Corresponding separation distances (*H*, in nm) are provided to the left of each image. The molecular orbital is plotted for iso-values of ±0.02 atomic units with orange and green denoting opposite signs. Spheres in red, blue, pink, and white represent Li^+, Na^+, phosphorus, and hydrogen respectively.

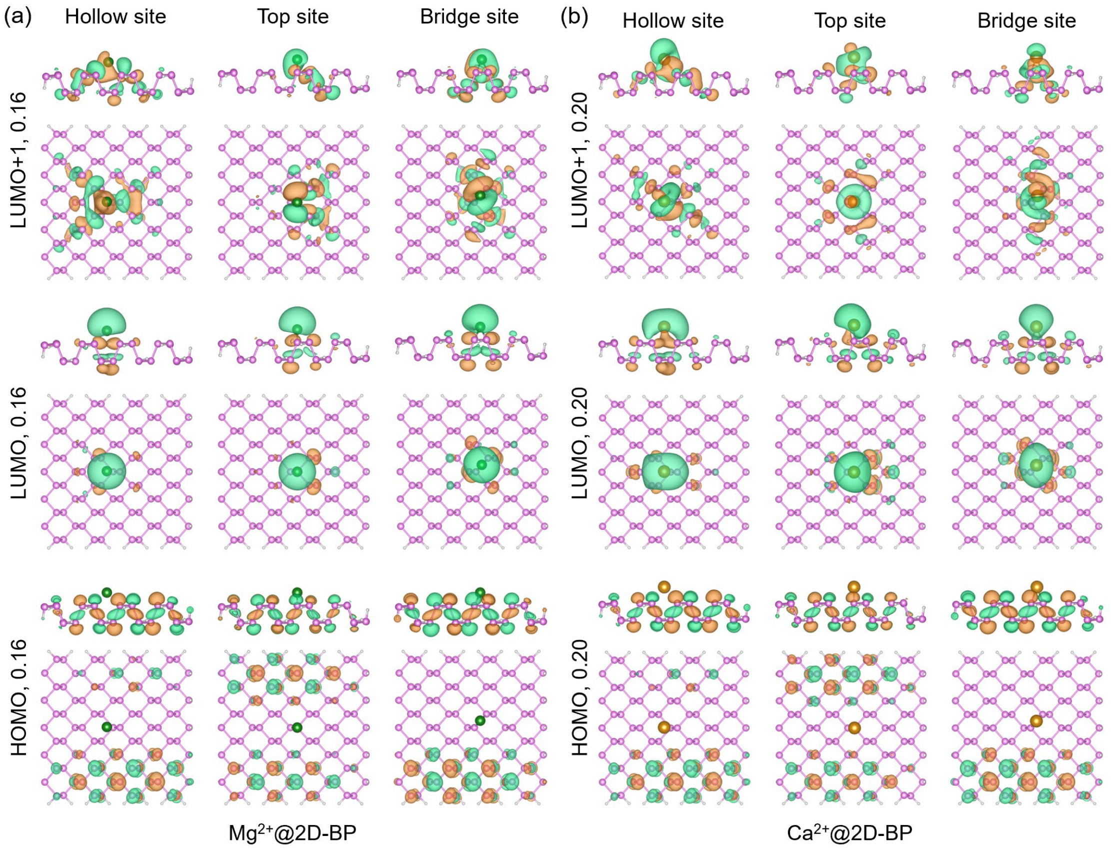

Figure S8. Spatial localization of frontier molecular orbitals in (a) Mg^{2+}@2D-BP and (b) Ca^{2+}@2D-BP complexes at the potential energy minimum across different adsorption sites (hollow, top, and bridge). Corresponding separation distances (*H*, in nm) are provided to the left of each image. The molecular orbital is plotted for iso-values of ±0.02 atomic units with orange and green denoting opposite signs. Spheres in green, orange, pink, and white represent Mg^{2+}, Ca^{2+}, phosphorus, and hydrogen respectively.
